# Boolean Feedforward Neural Network Modeling of Molecular Regulatory Networks for Cellular State Conversion

**DOI:** 10.3389/fphys.2020.594151

**Published:** 2020-12-01

**Authors:** Sang-Mok Choo, Laith M. Almomani, Kwang-Hyun Cho

**Affiliations:** ^1^Department of Mathematics, University of Ulsan, Ulsan, South Korea; ^2^Department of Bio and Brain Engineering, Korea Advanced Institute of Science and Technology (KAIST), Daejeon, South Korea

**Keywords:** molecular regulatory network, Boolean network modeling, feedforward neural networks, Boolean feedforward neural network, temporal data, cellular state conversion

## Abstract

The molecular regulatory network (MRN) within a cell determines cellular states and transitions between them. Thus, modeling of MRNs is crucial, but this usually requires extensive analysis of time-series measurements, which is extremely difficult to obtain from biological experiments. However, single-cell measurement data such as single-cell RNA-sequencing databases have recently provided a new insight into resolving this problem by ordering thousands of cells in pseudo-time according to their differential gene expressions. Neural network modeling can be employed by using temporal data as learning data. In contrast, Boolean network modeling of MRNs has a growing interest, as it is a parameter-free logical modeling and thereby robust to noisy data while still capturing essential dynamics of biological networks. In this study, we propose a Boolean feedforward neural network (FFN) modeling by combining neural network and Boolean network modeling approach to reconstruct a practical and useful MRN model from large temporal data. Furthermore, analyzing the reconstructed MRN model can enable us to identify control targets for potential cellular state conversion. Here, we show the usefulness of Boolean FFN modeling by demonstrating its applicability through a toy model and biological networks.

## Introduction

Cellular behavior is governed by intracellular molecular regulatory networks (MRNs), such as signaling and gene regulatory networks (Schmidt et al., 2005; Kim and Cho, 2006; Sreenath et al., 2008; Kim et al., 2011). Reconstruction and mathematical modeling of such MRNs based on biological experiments have been of great interest in the field of systems biology. Modeling MRNs has been, however, very challenging due to the limited availability of time course measurements from biological experiments. This can now be overcome by recent advancement of technologies in experimental data measurements, and thus, there is a growing interest in developing a new paradigm of modeling MRNs based on large data sets.

Single-cell technologies have emerged in the fields of genomics (Ludwig et al., 2019; Tritschler et al., 2019; Baslan et al., 2020; Yofe et al., 2020), epigenomics (Berkel and Cacan, 2019; Chen et al., 2019; Verma and Kumar, 2019), transcriptomics (Cui et al., 2019; He et al., 2020; Huang et al., 2020), proteomics (Minakshi et al., 2019; Zhu et al., 2019; Labib and Kelley, 2020), and metabolomics (Duncan et al., 2019; Kawai et al., 2019; Kumar et al., 2020). We can now obtain omics information of hundreds to thousands of individual cells from a single experiment. For instance, single-cell RNA sequencing technologies can measure messenger RNA concentration of hundreds to thousands of genes expressed by single cells, and single cell proteomics by mass spectrometry can quantify over 1,000 proteins per single cell at once (Budnik et al., 2018; Lun and Bodenmiller, 2020). Such single-cell data can be used as pseudo-time-series measurements of distinct cellular states that can provide a new opportunity for modeling MRNs.

There have been attempts to develop dynamic models of MRNs based on ordinary differential equations, regression models, and Boolean networks. Boolean models are more appropriate to be employed for modeling MRNs from pseudo-time-series single-cell data since high-throughput single-cell data are more noisy than conventional bulk sequencing data, and Boolean logical network models are relatively robust to noise. Constructing a Boolean network model usually requires two steps: generating pairs of Boolean input and output for each node in the MRN from states of pseudo-time-ordered single cells and then fitting the Boolean state update logic of each node to the data (Hamey et al., 2017). There are, however, a number of challenges in determining the backbone network structure and optimizing the regulatory logic to the measured data sets. To overcome such challenges, we propose an approach combining Boolean network modeling and feedforward neural network (FFN) learning algorithm, which is particularly useful for inferring input–output relationships from large temporal data. For this purpose, we use only temporal data of network nodes and do not need to determine the network structure nor to optimize the regulatory logics. Of note, in our Boolean FFN model, each node of MRN is represented by a single output node of an FFN with all MRN nodes as its input nodes, and then, the state transition dynamics of MRN can be simulated by executing the entire Boolean FFN model.

Considering a cellular state transition process, we can partition the temporal data of such a process into three parts: ordered pairs of initial cellular states, ordered pairs of transitional cellular states, and ordered pairs of final cellular states. These three ordered pairs can then be used for building initial, transitional, and final cellular states of FFNs, which can be referred to as iFFN, tFFN, and fFFN, respectively. Employing the trained iFFN, tFFN, and fFFN, we can generate trajectories starting from initial to terminal cellular states and use such state trajectories as new training data for building a cell fate transition FFN (cFFN) for each node.

The eventual goal of our study is to identify control targets that can induce desired cellular state conversion, and for this purpose, we propose to build cFFN using iFFN, tFFN, and fFFN based on temporal data measurements of network nodes. We demonstrate the effectiveness and possible application of the proposed Boolean FFN modeling of MRNs by applying it to a toy network model as well as real biological networks. In particular, we compare identified control targets for cellular state conversion between the Boolean FFN and its original Boolean network model in order to show the effectiveness of the proposed Boolean FFN modeling of MRNs.

## Results

### Overview of Constructing cFFN

**FIGURE 1:**
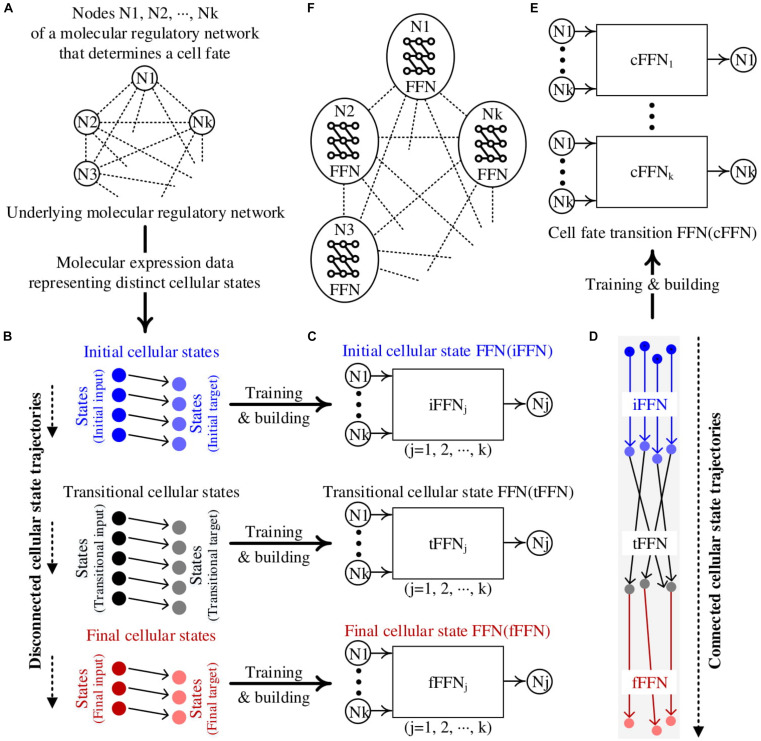
Construction of cell fate transition feedforward neural network (cFFN). **(A)** Nodes represent molecules of the hidden molecular regulatory network (MRN) that can be genes, proteins, or metabolites. **(B)** The measured molecular data representing distinct cellular states are partitioned: ordered pairs of initial, transitional, and final cellular states. Each circle denotes a Boolean state of k nodes for each cellular state. Each right-pointing arrow denotes the direction of updated state. There is no such arrow between two states in different partitions, resulting in disconnected cellular state trajectories. **(C)** The left and right states within the initial cellular states are referred to as initial input and target, respectively, to denote that they are used as training data for an FFN of node Nj. Here, the trained FFN is referred to as an iFFN for the node. Similarly, the left and right states in the transitional and final cellular states are used as training data to build tFFN and fFFN, respectively, for each node. **(D)** The application of iFFN, tFFN, and fFFN to the initial input, iFFN(initial input), and tFFN[iFFN (initial input)], respectively, generates connected trajectories from the initial states. **(E)** States upon each connected trajectory in **(D)** are used as training data for a cFFN. **(F)** A conceptual diagram illustrating our Boolean feedforward neural network modeling. Each circle denotes the constructed Boolean FFN for node Nj.

The overall procedure of constructing a cFFN is summarized in Figure 1. We presume that the nodes playing a significant role in the cellular state transition of interest are known, whereas the regulatory relationships among the nodes are unknown (Figure 1A). Here, all the nodes are assumed to have binarized values for their expression levels as to consider MRNs represented by Boolean network models. We also assume that marker nodes, which define specific desired or undesired states that are known, can be used as a primary basis for evaluation after identifying control targets for cellular state conversion.

We consider three clusters of Boolean states over the transition from initial to final states through transitional states, resulting in three sets of ordered pairs of initial, transitional, and final cellular states as shown in Figure 1B. These will also be referred to as the first, second, and third clusters to emphasize the order of cellular state transition. As we consider a transition process from an initial normal state to a final abnormal state, there is a tendency that the number of desired states decreases from the first to third clusters while the number of undesired states increases, which is referred to as marker tendency. In each cluster, the first state of ordered pair is assumed to be updated to the second state, which is represented by connecting arrows. However, there is no connection information between two clusters, resulting in no trajectory from initial to final states. We call these three consecutive clusters disconnected trajectories.

To construct connected trajectories, we build three FFNs, i.e., iFFN, tFFN, and fFFN, for each node using the corresponding cluster as training data (Figure 1C). The marker tendency is used as a constraint for training each FFN.

We consider the first states in the pairs of initial cellular states be the initial input. By applying iFFN, tFFN, and fFFN to each corresponding initial input as iFFN(initial input) and tFFN[iFFN(initial input)], we can construct connected trajectories from initial input to final output states (Figure 1D).

Using the set of states on each connected trajectory as new training data, we can construct cFFN for the node (Figure 1E). The entire MRN is then composed of cFFNs of the nodes within a network model, which is illustrated by a conceptual diagram in Figure 1F.

### Toy Network for Illustrating FFNs

#### Construction of cFFN

**FIGURE 2:**
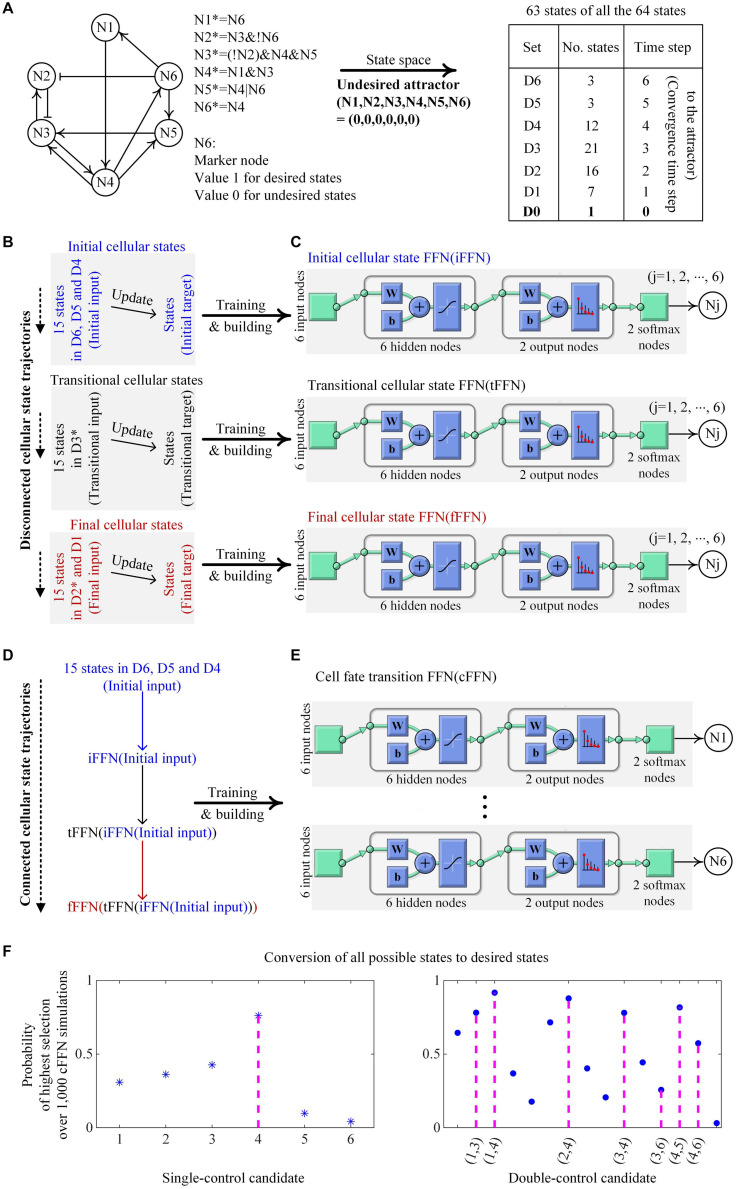
Construction of cFFN with training data generated by a toy network and its application for identifying control targets. **(A)** The toy network consists of nodes Nj (1 ≤ j ≤ 6) with its Boolean update logics. Nj and Nj* denote the Boolean states of Nj at time steps t and t + 1, respectively. Symbols &, |, and ! denote Boolean operators AND, OR, and NOT, respectively. Sharp and blunt arrows represent positive and negative effects, respectively, in the directed graph from Ni to Nj. Node N6 is assumed to be a unique marker, and thus, states of inactive or active N6 correspond to undesired or desired states, respectively. All possible states except one state of the network converge to an undesired state, which has all zero values and is designated as a unique undesired attractor. The 63 states are partitioned according to the converging time steps to the undesired attractor where D0 denotes a singleton set of the attractor and Dj denotes a set of states that will become attractors when they are updated j (1 ≤ j ≤ 6) times, referred to as converging time step j to the attractor. **(B)** Initial cellular states denote the states of 15 ordered pairs of which the first states are randomly chosen from D6 to D4 and their updated states become the second states. Transitional and final cellular states are defined similarly, where D3* and D2* denote the sets of all states in D3 and D2 except those states updated from the second initial and transitional states, respectively. Note that the updated states of initial and transitional states are not transitional and final states, respectively. **(C)** By employing the pattern recognition network (PatternNet) and using the training function (train) from Matlab, FFN for each node Nj is trained. The structure of FFN consists of six input nodes, one hidden layer of six nodes, output layer of two nodes, two ordered softmax nodes, and node Nj. Here, softmax nodes have values of the softmax function, and Nj has a value 1 if the value of the first softmax node is greater or equal to that of the second softmax node. The acronyms w and b denote weight and bias, respectively. Each of iFFN, tFFN, and fFFN consists of such six trained FFNs. **(D)** iFFN, tFFN, and fFFN are consecutively applied to the initial input, iFFN(initial input), and tFFN[iFFN(initial input)], and thereby connected cellular state trajectories are produced. **(E)** States upon each connected trajectory are used as training data for an FFN with the structure in **(C)**. The trained FFN is denoted as a cFFN. **(F)** Pinning the value of N4 to 1 is the only way to drive any states to desired ones. Here, N4 is the unique single-control target of value 1. Single-control candidates denote 6 nodes of pinned value 1 in the cFFN. The left panel shows the probability of each single-control candidate of value 1 to be a target of value 1. In this example, the value of Nj is fixed to 1 in the cFFN, and every possible state is updated accordingly. Then, the number of states driven to desired states is counted. Nj gets score 1 if the counted number is in the list of the two highest numbers of the candidates, or 0 otherwise. As a result, repeating this scoring process for each of 1,000 cFFNs gives the probability of Nj in the left panel, where N4 has the highest probability. In the right panel, seven ordered pairs (N1, N3), (N1, N4), (N2, N4), (N3, N4), (N3, N6), (N4, N5), and (N4, N6) of values (1, 1) are the only possible ways to drive any states to desired ones when fixing the values of two nodes to an ordered pair (1, 1). Here, the pairs are double-control targets of values (1, 1) and 15 pairs of two nodes are considered as double-control candidates of values (1, 1) in cFFN. This shows the probability of each double-control candidate of values (1, 1) to be a target of values (1, 1), where the scoring process is the same as that used in identifying single-control target by replacing the two highest numbers with the eight highest numbers.

We demonstrate an example of building iFFN, tFFN, fFFN, and cFFN using a toy network of six nodes with Boolean update logics to identify control targets in Figure 2. The graph in Figure 2A only represents collective regulatory relationships between two nodes in the network (without considering the regulatory logics), and node N6 is considered as a unique marker in this case, where a state is the desired state if N6 is active (value 1) or otherwise undesired (value 0) as shown in Figure 2A. All possible states except one state from the toy network converge to an undesired state, which is called an undesired attractor, and are partitioned into seven sets: D0 denotes a singleton set of the undesired attractor. Dj denotes those states converging to the attractor when they are updated j (1 ≤ j ≤ 6) times.

We use Dj to generate initial, transitional, and final cellular states as shown in Figure 2B. Fifteen states randomly chosen from D6, D5, and D4 and their one-time updated states are represented as the first and second states of ordered pairs of initial states, respectively. Fifteen states randomly chosen from D3^∗^ and their one-time updated states are represented as the first and second states of ordered pairs of transitional states, respectively. Here, D3^∗^ denotes the set of all states in D3 except those states that are updated from the second initial states. Fifteen states randomly chosen from D2^∗^ and D1 and their updated states are represented as the first and second states of ordered pairs of final states, respectively, where D2^∗^ denotes the set of all states in D2 except those states that are updated from the second transitional states (Supplementary Data 1).

The first and second states of ordered pairs of initial, transitional, and final states are used for training input and target of iFFN, tFFN, and fFFN, respectively, as shown in Figure 2C. The constraint of marker tendency is also considered when training each FFN (see section “Materials and Methods” for details). A sequential application of iFFN, tFFN, and fFFN to the initial input, iFFN(initial input), and tFFN[iFFN(initial input)], produces 15 trajectories as shown in Figure 2D. The two consecutive states on each trajectory are used as training input and target for a Boolean FFN, which is cFFN as shown in Figure 2E.

#### Conversion of Undesired States With cFFN

We demonstrate that cFFN can be used in identifying control targets for state conversion of undesired states to desired ones. Pinning the values of single node or two nodes during state update is referred to as single or double controls, respectively. To validate whether the control candidates identified from cFFN can drive the undesired states to desired ones, we compare the control “candidates” to control “targets” found by extensive simulation analysis of the original Boolean network models of MRNs.

##### Single-control target

To evaluate control candidates, we search for all single-control targets by simulating the Boolean network model of this toy network. For this particular example, when pinning the value of a node to 0 and updating every state according to the regulatory logics of the Boolean network model, there exists a state that cannot be driven to a desired state. This shows that there is no single-control target of value 0 in this case. However, there is a unique single-control target of value 1. Pinning the value of N4 to 1 is the only way to drive all possible states to desired states. This shows that N4 is a unique single-control target of value 1. To examine whether cFFN can be used to identify N4, we consider each node Nj (1 ≤ j ≤ 6) in cFFN as a single-control candidate of value 1.

To identify control candidates as the unique single-control target N4 by using cFFN, we define the probability of each single-control candidate of value 1 to be a single-control target of value 1. Here, the value of Nj is fixed to 1 in a given cFFN, and every possible state is updated using the cFFN. Then, the number of states driven to desired states is counted. After obtaining such counted numbers of all single-control candidates, Nj gets a score 1 if the counted number of Nj is one of the two highest numbers of the candidates, or 0 otherwise. Here, the number 2 is a kind of hyperparameter. We repeat this scoring process for each of 1,000 cFFNs and divide the total score of Nj by 1,000, which is represented as the probability of Nj shown in the left panel of Figure 2F. As a result, the single-control target N4 has the highest probability among all of the single-control candidates.

##### Double-control target

First, we performed a case study for double control by pinning the values of two nodes to (1, 1). We find that, if one of seven pairs, (N1, N3), (N1, N4), (N2, N4), (N3, N4), (N3, N6), (N4, N5), and (N4, N6), has pinned values as (1, 1), any states would eventually converge to desired states. As a result, those seven pairs are identified as double-control targets of values (1, 1). To examine whether cFFN can be used for identifying such double-control targets of values (1, 1), we consider 15 pairs of two nodes as double-control candidates of values (1, 1) and evaluate each of them. To identify control candidates for the double-control targets of values (1, 1) by using cFFN, the probability of each double-control candidate of values (1, 1) to be a target of values (1,1) is defined similarly to that used in the case of single-control candidate. This can be done by replacing single control and the two highest numbers with double control and the eight highest numbers, respectively. We present the probability in the right panel of Figure 2F. We find that five of the seven double-control targets of values (1,1) are in the list of five highest probabilities.

We performed the second case study for double control by pinning the values of two nodes to values (0, 1) since there is no double-control target of values (0, 0). If one of five ordered pairs, (N1, N4), (N2, N4), (N3, N4), (N5, N4), and (N6, N4), has values (0, 1), then any states would eventually converge to the desired states. As a result, those five pairs are identified as double-control targets of values (0, 1). To examine whether cFFN can be used for identifying such five double-control targets of values (0,1), we consider 30 ordered pairs of two nodes in cFFN as double-control candidates of values (0, 1) and evaluate each of them. The probability of each double-control candidate of values (0, 1) to be a target of values (0, 1) is defined similarly to that used in the case of double-control candidate of values (1, 1) by replacing the eight highest numbers with the 10 highest numbers. We present the probability in Supplementary Figure 1, where all the five double-control targets of values (0, 1) have the five highest probabilities.

### Applications of FFN for Identifying Biomolecular Control Targets

**FIGURE 3:**
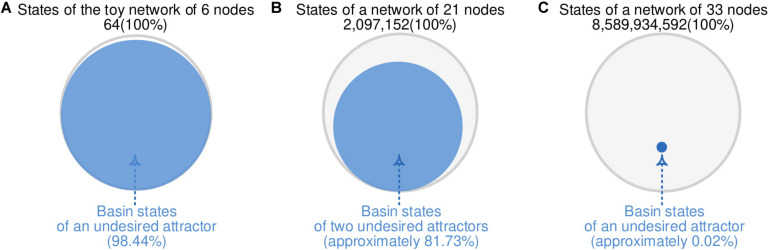
Different portion of states converging to undesired attractors. **(A)** A total of 63 of 64 states from the toy network with six nodes in Figure 2 converges to an undesired attractor, where the 63 states are referred to as basin states of the undesired attractor with 98.44% basin size. **(B)** A reduced colitis-associated colon cancer network model with 21 nodes in Figure 4 has 81.73% basin size. **(C)** A mitogen-activated protein kinase network model with 33 nodes in Figure 5 has 0.02% basin size.

To construct cFFN of an MRN and demonstrate its applicability for identifying control targets as in Figure 3A, we employ two biomolecular network models. One of the network models is composed of 21 nodes and has a large portion (81.73%) of states converging to an undesired state. In contrast, the other network model is composed of 33 nodes and has a unique undesired state with a very small portion (0.02%) of states converging to an undesired state.

#### Colitis-Associated Colon Cancer Network

##### Construction of cFFN

**FIGURE 4:**
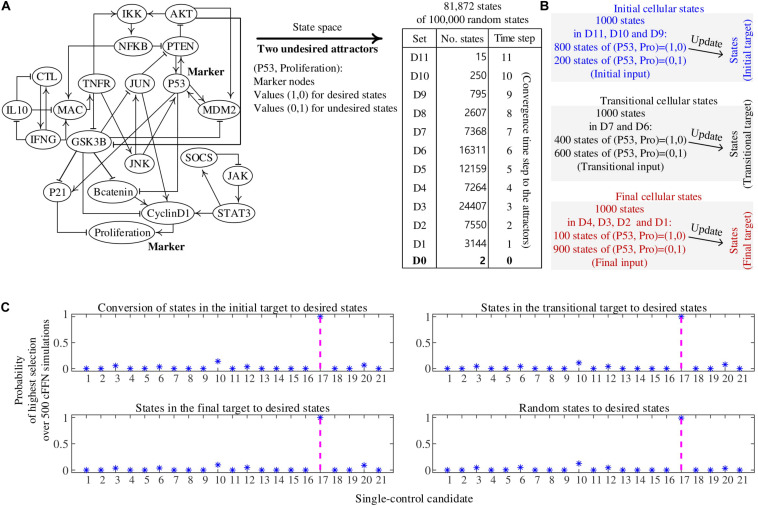
Construction of cell fate transition feedforward neural network (cFFN) with training data generated by a biomolecular network and its application to identifying single-control targets. **(A)** A reduced colitis-associated colon cancer network of 21 nodes (CACC21). Nodes P53 and Proliferation are considered as markers for desired and undesired states, respectively. Its update logics and two undesired attractors are shown in Supplementary Data 2. We generate 100,000 random states, of which 81,872 states converge to one of the undesired attractors and are partitioned into Dj (1 ≤ j ≤ 11). Dj denotes a set of states that will transit into one of the attractors when they are updated j times, which is referred to as converging time step j to the attractors. D0 denotes a set of the two undesired attractors. **(B)** The 1,000 states in the initial cellular states denote 1,000 randomly generated states from D11, D10, and D9. Here, 800 states have (P53, Proliferation) = (1,0) and 200 states have (P53, Proliferation) = (1,0), which are referred to as initial input for training iFFN. The states are updated one time, where the updated states are referred to as initial target for training iFFN. The 1,000 states in the transitional cellular states denote 1,000 randomly generated states from D7 and D6. Here, 400 states have (P53, Proliferation) = (1,0) and 600 states have (P53, Proliferation) = (1,0), which are referred to as transitional input for training tFFN. The transitional target denotes one-time updated states in the transitional input. The 1,000 states in the final cellular states denote 1,000 randomly generated states from D4, D3, and D2. Here, 100 states have (P53, Proliferation) = (1,0) and 900 states have (P53, Proliferation) = (1,0), which are referred to as final input for training fFFN. Similarly, final target is defined by using final input. **(C)** Four subplots show the probability of each single-control candidate of value 1 to be a single-control target, where 500 cFFNs and the five highest numbers are used in the scoring process. The states in the initial, transitional, and final targets and random states are used in the scoring process. As a result, the unique single-control target N17 of value 1 has the highest probability.

The biomolecular network in Figure 3B is a reduced colitis-associated colon cancer network of 21 nodes Nj (1 ≤ j ≤ 21) shown in Figure 4A, which is denoted by CACC21 (Lu et al., 2015). Node ID Nj and the state update logics of CACC21 are provided in Supplementary Data 2. Markers for desired and undesired states are P53 and Proliferation nodes; states with values (P53, Proliferation) = (1, 0) and (0, 1) are considered as desired and undesired states, respectively. Here, P53 and Proliferation are molecular marker nodes indicating programmed cell death/arrest and uncontrolled cell growth, respectively. In this network, 81.73% of all possible states converge to one of two undesired attractors (Choo et al., 2019 and Supplementary Data 2). When we generate 100,000 random states, 81,870 states converge to one of the undesired attractors at time steps from 1 to 11, which are partitioned into 11 sets of Dj (1 ≤ j ≤ 11). D0 denotes a set of the two undesired attractors.

Initial cellular states are 1,000 states randomly chosen from D11 to D9 and their one-time updated states, which are referred to as the first and second states of ordered pairs of initial states, respectively. Here, 800 and 200 states of the first states have values (P53, Proliferation) = (1, 0) and (0, 1), respectively, in Figure 4B. Transitional cellular states are 1,000 states randomly chosen from D7 to D6 and their one-time updated states, referred to as the first and second states of ordered pairs of transitional states, respectively. Here, 400 and 600 states of the first states have values (P53, Proliferation) = (1, 0) and (0, 1), respectively. Final cellular states are 1,000 states randomly chosen from D4 to D1 and their updated states, referred to as the first and second states of ordered pairs of final states, respectively. Here, 100 and 900 states of the first states have values (P53, Proliferation) = (1, 0) and (0, 1), respectively.

The first and second states of ordered pairs of initial, transitional, and final states are used as training input and target for iFFN, tFFN, and fFFN, respectively. Restrictions of marker tendency are added for training each FFN (see section “Materials and Methods”). The consecutive application of iFFN, tFFN, and fFFN to the initial input, iFFN(initial input), and tFFN[iFFN(initial input)], respectively, produces 1,000 trajectories that are then used as training data for cFFN.

##### Single-control target

To validate whether the control candidates identified from cFFN can drive undesired states to desired ones, we compare the control “candidates” to the control “targets” found by extensive simulation analysis of CACC21. We search for all single-control targets by simulating the Boolean network model of CACC21.

There exists no node that can be driven to a desired state when pinning the value of the node to 0 and updating every state according to the regulatory logics of CACC21; there is no single-control target of value 0 in this case. However, there is a unique single-control target of value 1. Pinning the value of N17 (PTEN) to 1 is the only unique single-control target of value 1 that can drive 100 sets of 1,000 random states to desired states. To examine whether cFFN can be used to identify this unique target, we consider each node Nj (1 ≤ j ≤ 21) in cFFN as a single-control candidate of value 1.

To identify control candidates as the unique single-control target N17 by using cFFN, we define the probability of each single-control candidate Nj of value 1 to be a single-control target of value 1. Here, the value of Nj is fixed to 1 in a given cFFN, and 1,000 states of the initial target are updated 100 times using the cFFN. Then, the number of states in the initial target driven to desired states is counted. After obtaining the counted numbers of all single-control candidates of value 1, Nj gets a score 1 if its counted number is one of the five highest numbers of the candidates, or 0 otherwise. We repeat the scoring process for each of 500 cFFNs and divide the total score of Nj by 500, which gives the probability of Nj shown in the upper left panel of Figure 4C. Moreover, the initial target used in the scoring process is a hyperparameter. Thus, we replace it with transitional, final, and random states and present the probability of single-control candidates in the upper right, bottom left, and bottom right panels of Figure 4C, respectively. As a result, the unique single-control target N17 has also the highest probability.

##### Double-control target

We find 22 and 30 double-control targets of values (1, 1) and (0, 1), respectively, from extensive simulation analysis of the Boolean network model just as the case of single-control targets. We find that there is a unique double-control target of values (0, 0). Pinning the values of (N8, N13) to (0, 0) is the only way to drive all possible states to desired states, where N8 = IL10 and N13 = MDM2. Hence (N8, N13), is a unique double-control target of values (0, 0). To examine whether cFFN can be used to identify this target, we consider 210 pairs of two nodes as double-control candidates of values (0, 0) and evaluate each of them. To identify control candidates for the double-control targets by using cFFN, we define the probability of each double-control candidate (Nj, Nk) (1 ≤ j < k ≤ 21) of values (0, 0) to be a unique double-control target of values (0, 0). Here, the values of a double-control candidate (Nj, Nk) are fixed to (0, 0) in a given cFFN, and 1,000 states in the final target are updated 100 times by using the cFFN. Then, the number of states driven to desired states is counted. The number of states within the final target driven to desired states is counted for each of 500 cFFNs. After obtaining the counted numbers of all double-control candidates of values (0, 0), (Nj, Nk) gets a score 1 if its counted number is within the top 20% over the total of 210, or 0 otherwise. We repeat this scoring process for each of 500 cFFNs and divide the total score of Nj by 500, which gives the probability of (Nj, Nk) shown in Supplementary Figure 2. It shows that the double-control target (N8, N13) of values (0, 0) is within the top 26 out of all 210 probabilities. Moreover, we further test by changing the percentile from top 20% with 30 and 40% and find that the double-control target (N8, N13) of values (0, 0) is within the top 16 and 17, respectively, as shown in Supplementary Figure 2.

#### Mitogen-Activated Protein Kinase Network

##### Construction of cFFN

**FIGURE 5:**
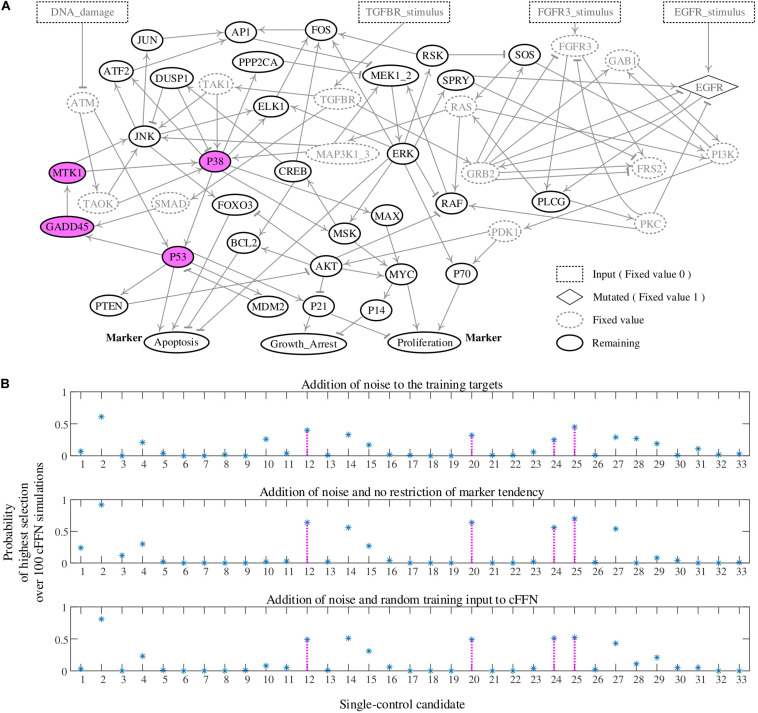
Construction of cell fate transition feedforward neural network (cFFN) with noisy training data generated by a biomolecular network of 33 nodes and application of cFFN for identifying single-control targets of value 1. Node ID Nj (1 ≤ j ≤ 33) is provided in Supplementary Data 3. **(A)** A mitogen-activated protein kinase network of 53 nodes. Its update logics are provided in Supplementary Data 3. Rectangle and diamond nodes have fixed values of 0 and 1 in the update logics, respectively, which lead to fixed values of the dotted circles. All nodes except the nodes having fixed values are 33 nodes, which are represented by solid circle nodes. The network of 33 nodes is called MAPK33. Update logics of these nodes are provided in Supplementary Data 3. If one of four, N12, N20, N24, and N25, has the pinned value 1, any state within 2,000 sets of 1,000 random states would eventually converge to a desired state, where states in 1,000 sets are randomly chosen from all the states converging to the undesired attractor. The four red nodes denote the four single-control targets of value 1. **(B)** In the upper panel, the probability of single-control candidate Nj to be a single-control target of value 1 is calculated using the noisy training targets for iFFN, tFFN, and fFFN. Here, restrictions of marker tendency and the number of noisy fourth states driven to desired states are used. The four single-control targets of value 1 are ranked as the third, fifth, ninth, and second in descending order of probability. In the middle, the probability is calculated using the noisy training targets for iFFN, tFFN, and fFFN. Moreover, no restrictions of marker tendency and 1,000 states at time step 1 to the undesired attractor instead of the noisy fourth states are used. As a result, the four single-control targets are ranked as the third, third, fifth, and second in descending order of probability. In the bottom panel, the probability is calculated using noisy training targets for iFFN, tFFN, and fFFN, and random states converging to the undesired attractor and the noisy first states instead of the first states and the noisy fourth states, respectively. Finally, the four single-control targets are ranked as the fifth, fifth, third, and second in descending order of probability.

The second biomolecular network in Figure 3C is a mitogen-activated protein kinase network composed of 53 nodes as depicted in Figure 5A with their update logics in Supplementary Data 3 (Grieco et al., 2013). The values of epidermal growth factor receptor (EGFR) and all the input nodes are fixed to 1 (dotted diamond in Figure 5A) and 0 (dotted rectangles in Figure 5A), respectively. As a result, 15 nodes have fixed values (dotted circles in Figure 5A). The remaining 33 nodes (solid circles in Figure 5A) form a subnetwork, which is called MAPK33. The state update logics of MAKP33 are provided in Supplementary Data 3. Nodes of Apoptosis and Proliferation are considered as markers for desired and undesired states; states with (Apoptosis, Proliferation) = (1, 0) and (0, 1) are considered as desired and undesired states, respectively. Here, Apoptosis denotes programmed cell death. About 0.02% out of all possible states converge to an undesired attractor in Supplementary Data 3 and 4 (Choo et al., 2019).

We generate 1,000 random initial states (first states) converging to the undesired attractor, where 900 and 100 states have active Apoptosis and Proliferation, respectively. The first states are updated one time to the next by using the update logics. Similarly, the second states are updated one time to the third states, which are then also updated to the fourth states. To demonstrate the general applicability of cFFN in identifying control targets, the MAPK33 is used in different ways from the toy network and CACC21 as follows: we introduce noise to the second states by changing the values of six randomly chosen nodes in each of the second states, which are referred to as noisy second states. Similarly, noisy third and fourth states are defined. Therefore, (1st, noisy 2nd), (2nd, noisy 3rd), and (3rd, noisy 4th) are training data for iFFN, tFFN, and fFFN, respectively, and restrictions of marker tendency are added for training each FFN (see section “Materials and Methods” for details). Applying the trained iFFN, tFFN, and fFFN3 to the first states, iFFN(1st states) and tFFN[iFFN1(1st states)], respectively, result in 1,000 connected trajectories from the first states of which are then used to train a cFFN.

##### Single-control target

To evaluate the control candidates, we search for all single-control targets by simulating the Boolean network model of MAPK33. The first case of single control is pinning the value of one node to 1. There exist four single-control targets of value 1. We find that, if one of four, N12 (GADD45), N20 (MTK1), N24 (P38), and N25 (P53), has the pinned value 1, any state among 2,000 sets of 1,000 random states would eventually converge to a desired state. Here, the states in 1,000 sets are randomly chosen from all the states converging to the undesired attractor. Node ID Nj is provided in Supplementary Data 3. As a result, those four nodes are single-control targets of value 1, which are shown as red nodes in Figure 5A. To examine whether cFFN can be used to identify these single-control targets of pinning value 1, we consider each node Nj (1 ≤ j ≤ 33) in cFFN as a single-control candidate of value 1.

To identify control candidates as a single-control target by using cFFN, we define the probability of single-control candidate Nj of value 1 to be a single-control target of value 1. Here, the value of Nj is fixed to 1 in a given cFFN, and the 1,000 noisy fourth states are updated 100 times using the cFFN. Then, the number of noisy fourth states driven to desired states is counted. After obtaining such counted numbers of all single-control candidates, Nj gets a score 1 if the counted number of Nj is 1 of the 10 highest numbers among the candidates, or 0 otherwise. We repeat the scoring process for each of 100 cFFNs and divide the total score of Nj by 100, which gives the probability of Nj shown in the upper panel of Figure 5B. As a result, the four single-control targets of value 1 are ranked as the third, fifth, ninth, and second in descending order of probability.

The middle panel of Figure 5B shows the probability of each single-control candidates to be a single-control target of value 1 without introducing the restriction of marker tendency. In addition, 1,000 states at converging time step 1 to the undesired attractor are used instead of the noisy fourth states driven to desired states in the upper panel of Figure 5B. As a result, the four single-control targets are ranked as the third, third, fifth, and second in descending order of probability. Finally, the bottom panel in Figure 5B shows the probability of each single-control candidates to be a single-control target of value 1. This can be done by using 1,000 random states converging to the undesired attractor and the noisy second states, instead of the first states and the noisy fourth states in the upper panel of Figure 5B, respectively. As a result, the four single-control targets are ranked as the fifth, fifth, third, and second in descending order of probability.

The second case of single control is pinning the value of one node to value 0. We find that if one of two nodes, N6 (CREB) and N7 (DUSP1), has value 0, then any state within 100 sets of 1,000 random states converging to the unique undesired attractor would eventually converge to desired states. Hence, the two nodes are single-control targets of value 0. We define the probability of single-control candidate Nj to be a single-control target of value 0 as in the upper panel of Figure 5B. As a result, the two single-control targets of value 0 have the first two highest probabilities as shown in Supplementary Figure 3.

## Discussion

Owing to the recent development of high throughput single cell measurement technologies, various omics data are now becoming more available that can be used for quantifying gene or protein expressions of hundreds to thousands of cells at a time. Those data can be ordered according to pseudo-time axis, and then, we can use them to investigate dynamic processes in cellular state transitions such as differentiation and tumorigenesis. One most important application of such data is developing a mathematical model of the MRN within a cell since it determines cellular dynamic behaviors. Boolean network models have been actively studied, as they are parameter-free logical dynamic models that can still represent many essential dynamics of MRNs and are also robust to noise contained in the data used for logic fitting. All previous studies on developing Boolean network models have focused on inferring the backbone network structures and optimizing the regulatory logical rules among the nodes of MRNs. In this study, we proposed a totally different approach by representing each node (i.e., molecule) of MRNs by a single output node of an FFN and then fitted the whole MRN composed of as many FFNs as the number of nodes to the measured pseudo-time course data such that the resulting Boolean FFN can reproduce all the predicted molecular expression levels of nodes for any initial state values. In this approach, we do not need to determine the regulatory network structure in advance, as it is obtained as a result of learning. To use our method, we only need to know (or determine) *a priori* the nodes that constitute the regulatory network. Then, we can apply our method based on the temporal measurement data of the network nodes. It is also remarkable that the proposed Boolean FFN modeling is quite robust to noise in the training data.

To show validity and applicability of the proposed Boolean FFN modeling, we considered three different network examples and further applied our method to identify control targets that can induce cellular state conversion to desired ones. We found that our method can accurately identify all those control targets that are revealed by extensive simulation analysis of the original dynamic network models. For the toy example network and CACC21 network, three clusters of states that are sequentially ordered upon the pseudo-time course trajectory leading to undesired attractors are generated by dividing the state transition trajectory into initial, middle, and final clusters of states. The first cluster is a set of states at early time steps, where ordered pairs of the states and their updated states are used as training data for iFFN. Similarly, the second and third clusters are defined and used as training datasets for tFFN and fFFN, respectively. Finally, using the first cluster and three FFNs, connected trajectories are constructed and the states on which are used as training data for cFFN. We used an ensemble of cFFNs to identify control targets for cellular state conversion, which shows general applicability of the proposed Boolean FFN modeling to biological network control for state conversion. Identifying control targets is important for cell fate control toward a desired cellular state. For instance, we can consider a state conversion from a malignant cancerous state to a benign normal state, which is called cancer reversion (Cho et al., 2016, 2017; Choi et al., 2020; Lee et al., 2020). We also showed that our method is robust to noisy data through the example of MAPK33 network.

Three Boolean FFNs (iFFN, tFFN, and fFFN) are used only to generate training data for building cFFN, but they can also be used to identify control targets for cellular state conversion among the initial, transitional, and final cellular states. Nevertheless, the key aspect of our proposed framework does not lie in the concept of iFFN, tFFN, and fFFN but in combining neural network modeling and Boolean network modeling approaches. In other words, we can use temporal data as training data for directly building a Boolean FFN without building iFFN, tFFN, and fFFN. Note that we investigated the attractor of a Boolean network only to generate temporal data, so our framework can be used without searching for attractors if temporal data of a cellular state transition process are given. To demonstrate the applicability of our framework without building such three Boolean FFNs and searching for an attractor, we employed the actually measured pseudo-time course single-cell data over the progression from hematopoietic stem cells toward lymphoid-primed multipotent progenitors (Hamey et al., 2017) and directly built a Boolean FFN using these pseudo-time data. From the FFN, we could identify an optimal single-control candidate as shown in Supplementary Figure 4. It remains as a future study for experimentally validating this result. Our future study also includes applying the proposed framework to identifying control candidates for cancer reversion together with its experimental validation.

We note that the proposed method might fail to identify optimal control targets if the training data are randomly chosen from a set of small portion of states having a property converging to an undesired attractor. We also note that there are many hidden hyperparameters to be determined in our proposed modeling framework since we employed a machine learning algorithm, FFN. For instance, we used only one hidden layer for the structure of FFN, and the number of hidden nodes was simply set to the number of molecules in the MRN. Although the structure is very simple, it worked well for the temporal data obtained from both the toy model and biological networks. However, different structures and hyperparameter values might be chosen for temporal data from other biological networks. The proposed Boolean FFN modeling framework of MRNs is universal, and thus, it can be applied to various types of molecular data obtained across state transitions.

## Materials and Methods

### Building Feedforward Neural Networks With Matlab

Let us consider an MRN represented by a Boolean network model. Here, we propose a new approach for modeling each node *x*_*i*_(1≤*i*≤*k*) of the Boolean network model using FFN. For this, we assume that three sets of ordered pairs of initial, transitional, and final states are measured over a dynamic process of state transition, which are denoted by *PI*, *PT*, and *PF*, respectively. These are defined as follows:

$$\begin{array}{c} P\,I=\left\{\left(s_{i\,n,n}^{I},s_{t\,a\,r,n}^{I}\right)|1\leq n\leq I_{k},\,\left\{s_{i\,n,n}^{I},s_{t\,a\,r,n}^{I}\right\}\subset {\left\{0,1\right\}}^{k}\right\},\\ P\,T=\left\{\left(s_{i\,n,n}^{T},s_{t\,a\,r,n}^{T}\right)|1\leq n\leq T_{k},\,\left\{s_{i\,n,n}^{T},s_{t\,a\,r,n}^{T}\right\}\subset {\left\{0,1\right\}}^{k}\right\},\\ P\,F=\left\{\left(s_{i\,n,n}^{F},s_{t\,a\,r,n}^{F}\right)|1\leq n\leq F_{k},\,\left\{s_{i\,n,n}^{F},s_{t\,a\,r,n}^{F}\right\}\subset {\left\{0,1\right\}}^{k}\right\}. \end{array}$$

where *PI* has *I*_*k*_ ordered pairs $\left(s_{i\,n,n}^{I},s_{t\,a\,r,n}^{I}\right)$ for 1≤*n*≤*I*_*k*_ such that $s_{i\,n,n}^{I}$ and $s_{t\,a\,r,n}^{I}$ are Boolean states of nodes *x*_*i*_(1≤*i*≤*k*). The symbols *n* and *I* in $\left(s_{i\,n,n}^{I},s_{t\,a\,r,n}^{I}\right)$ denoted that $\left(s_{i\,n,n}^{I},s_{t\,a\,r,n}^{I}\right)$ is an *n*th pair of *PI*. The symbols *in* and *tar* in $\left(s_{i\,n,n}^{I},s_{t\,a\,r,n}^{I}\right)$ denoted that $s_{i\,n,n}^{I}$ and $s_{t\,a\,r,n}^{I}$ are elements of input and target for training an FFN, respectively. The symbols in defining the terms, *PT* and *PF*, are similar to that of*PI*.

#### Construction of an FFN With Training Data ***PI*** (iFFN)

Using the Matlab function “patternnet,” we construct the structure of FFN for a node *x*_*i*_ with input, one hidden layer, one output layer of two nodes, two softmax nodes and node *x*_*i*_. The value of *x*_*i*_ is 1 if the value of the first softmax node out of two is greater than or equal to that of the second node. The FFN for node *x*_*i*_ is trained with input $s_{i\,n}^{I}=\left(s_{i\,n,1}^{I},\cdots ,s_{i\,n,I_{k}}^{I}\right)$ and target $s_{t\,a\,r,x_{i}}^{I}=\left(s_{t\,a\,r,1,x_{i}}^{I},\cdots ,s_{t\,a\,r,I_{k},x_{i}}^{I}\right)$ by using the Matlab function “train:”

$s_{t\,a\,r,\ell ,x_{i}}^{I}=\left(s_{t\,a\,r,\ell ,x_{i},1}^{I},\,s_{t\,a\,r,\ell ,x_{i},2}^{I}\right)$ such that

st⁢a⁢r,ℓ,xi,1I={1if⁢xi⁢has⁢value⁢ 1⁢in⁢st⁢a⁢r,ℓI,0otherwise,st⁢a⁢r,ℓ,xi,2I=1-st⁢a⁢r,ℓ,xi,1I.

The sizes of input and target are *k*×*I*_*k*_ and 2×*I*_*k*_, respectively. For training, we use the classification threshold of 0.6 and add restrictions that the FFN can satisfy the marker tendency, which is described in detail in section “Marker Tendency.” The trained FFN for node *x*_*i*_ is called “$F\,F\,N_{x_{i}}^{1}$.” Then, we can use a vector-valued function notation as follows:

$i\,F\,F\,N=\left(F\,F\,N_{x_{1}}^{1},\cdots ,F\,F\,N_{x_{k}}^{1}\right)$ and simply *F*^1^ = *iFFN*.

Then, the output of *F*^1^ can be written as follows:

$$F^{1}\,\left(s_{i\,n}^{I}\right)=\left\{\left(F\,F\,N_{x_{1}}^{1}\,\left(s\right),\cdots ,F\,F\,N_{x_{k}}^{1}\,\left(s\right)\right)|s\in s_{i\,n}^{I}\right\},$$

where $F\,F\,N_{x_{i}}^{1}\,\left(s\right)$ denotes the value of *x*_*i*_ obtained by substituting a state $s\in s_{i\,n}^{I}$ into *FFN*_*x_i_*_^1^.

#### Construction of an FFN With Training Data ***PT*** (tFFN)

Using the Matlab function “patternnet,” we construct the structure of FFN for a node *x*_*i*_ with input, one hidden layer, one output layer of two nodes, two softmax nodes, and node *x*_*i*_. The value of *x*_*i*_ is 1 if the value of the first softmax node out of two is greater than or equal to that of the second node. The FFN for node *x*_*i*_ is trained with input $s_{i\,n}^{T}=\left(s_{i\,n,1}^{T},\cdots ,s_{i\,n,T_{k}}^{T}\right)$ and target $s_{t\,a\,r,x_{i}}^{T}=\left(s_{t\,a\,r,1,x_{i}}^{T},\cdots ,s_{t\,a\,r,T_{k},x_{i}}^{T}\right)$ by using the Matlab function “train:”

$s_{t\,a\,r,\ell ,x_{i}}^{T}=\left(s_{t\,a\,r,\ell ,x_{i},1}^{T},\,s_{t\,a\,r,\ell ,x_{i},2}^{T}\right)$ such that

st⁢a⁢r,ℓ,xi,1T={1if⁢xi⁢has⁢value⁢ 1⁢in⁢st⁢a⁢r,ℓT,0otherwise,st⁢a⁢r,ℓ,xi,2T=1-st⁢a⁢r,ℓ,xi,1T.

The sizes of input and target are *k*×*T*_*k*_ and 2×*T*_*k*_, respectively. For training, we use the classification threshold of 0.6 and add restrictions that the FFN can satisfy the marker tendency. The trained FFN is called “$F\,F\,N_{x_{i}}^{2}$.” Then, we can use a vector-valued function natation as follows:

$t\,F\,F\,N=\left(F\,F\,N_{x_{1}}^{2},\cdots ,F\,F\,N_{x_{k}}^{2}\right)$ and simply *F*^2^ = *tFFN*.

#### Construction of an FFN With Training Data ***PF*** (fFFN)

Using the Matlab function “patternnet,” we construct the structure of FFN for a node *x*_*i*_ with input, one hidden layer, one output layer of two nodes, two softmax nodes, and node *x*_*i*_. The value of *x*_*i*_ is 1 if the value of the first softmax node out of two is greater than or equal to that of the second node. The FFN for node *x*_*i*_ is trained with input $s_{i\,n}^{F}=\left(s_{i\,n,1}^{F},\cdots ,s_{i\,n,F_{k}}^{F}\right)$ and target $s_{t\,a\,r,x_{i}}^{F}=\left(s_{t\,a\,r,1,x_{i}}^{F},\cdots ,s_{t\,a\,r,F_{k},x_{i}}^{F}\right)$ by using the Matlab function “train:”

$s_{t\,a\,r,\ell ,x_{i}}^{F}=\left(s_{t\,a\,r,\ell ,x_{i},1}^{F},\,s_{t\,a\,r,\ell ,x_{i},2}^{F}\right)$ such that

st⁢a⁢r,ℓ,xi,1F={1if⁢xi⁢has⁢value⁢ 1⁢in⁢st⁢a⁢r,ℓF,0otherwise,st⁢a⁢r,ℓ,xi,2F=1-st⁢a⁢r,ℓ,xi,1F.

The sizes of input and target are *k*×*F*_*k*_ and 2×*F*_*k*_, respectively. For training, we use the classification threshold of 0.6 and add restrictions that the FFN can satisfy the marker tendency. The trained FFN is called “$F\,F\,N_{x_{i}}^{3}$.” Then, we can use a vector-valued function notation as follows:

$f\,F\,F\,N=\left(F\,F\,N_{x_{1}}^{3},\cdots ,F\,F\,N_{x_{k}}^{3}\right)$ and simply *F*^3^ = *fFFN*.

#### Construction of a Cell Fate Transition FFN

We call the following set as “connected trajectories:”

$$\{\left(s_{1},s_{2},s_{3},s_{4}\right)|\left(s_{1},s_{2},s_{3},s_{4}\right)\in s_{i\,n}^{I}\times F^{1}\left(s_{i\,n}^{I}\right)\times F^{2}\left(F^{1}\left(s_{i\,n}^{I}\right)\right)\times F^{3}\left(F^{2}\left(F^{1}\left(s_{i\,n}^{I}\right)\right)\right).\},$$

where the symbol ∈ represents that *s*_1_, *s*_2_, *s*_3_ and *s*_*4*_ are one of states$s_{i\,n,n}^{I},\,F\,F\,N_{x_{i}}^{1}\,\left(s_{i\,n,n}^{I}\right),\,F\,F\,N_{x_{j}}^{2}\,\left(F\,F\,N_{x_{i}}^{1}\,\left(s_{i\,n,n}^{I}\right)\right)$, and $F\,F\,N_{x_{\ell }}^{3}\,\left(F\,F\,N_{x_{j}}^{2}\,\left(F\,F\,N_{x_{i}}^{1}\,\left(s_{i\,n,n}^{I}\right)\right)\right)$ for 1≤*n*≤*I*_*k*_ and 1≤*x*_*i*_,*x*_*j*_,*x*_ℓ_≤*k*, respectively. An FFN for node *x*_*i*_ is trained with input $s_{i\,n}^{c\,F\,F\,N}=\left(s_{i\,n}^{I},\,F^{1}\,\left(s_{i\,n}^{I}\right),\,F^{2}\,\left(F^{1}\,\left(s_{i\,n}^{I}\right)\right)\right)$ and target

$$s_{t\,a\,r,x_{i}}^{c\,F\,F\,N}=\left(F^{1}\,{\left(s_{i\,n}^{I}\right)}_{x_{i}},\,F^{2}\,{\left(F^{1}\,\left(s_{i\,n}^{I}\right)\right)}_{x_{i}},\,F^{3}\,{\left(F^{2}\,\left(F^{1}\,\left(s_{i\,n}^{I}\right)\right)\right)}_{x_{i}}\right)$$

by using the Matlab function “train.” Here,

$F^{1}\,{\left(s_{i\,n}^{I}\right)}_{x_{i}}=\left(F^{1}\,{\left(s_{i\,n,1}^{I}\right)}_{x_{i}},\cdots ,F^{1}\,{\left(s_{i\,n,I_{k}}^{I}\right)}_{x_{i}}\right)$ such that

$F^{1}\,{\left(s_{i\,n,n}^{I}\right)}_{x_{i}}=\left(F^{1}\,{\left(s_{i\,n,n}^{I}\right)}_{x_{i},1},F^{1}\,{\left(s_{i\,n,n}^{I}\right)}_{x_{i},2}\right)$ and

F1⁢(si⁢n,nI)xi,1={1if⁢xi⁢has⁢value⁢ 1⁢in⁢F1⁢(si⁢n,nI),0otherwise,F1⁢(si⁢n,nI)xi,2=1-F1⁢(si⁢n,nI)xi,1.

The remaining symbols in $F^{2}\,{\left(F^{1}\,\left(s_{i\,n}^{I}\right)\right)}_{x_{i}}$ and $F^{3}\,{\left(F^{2}\,\left(F^{1}\,\left(s_{i\,n}^{I}\right)\right)\right)}_{x_{i}}$are similarly defined as those in $F^{1}\,{\left(s_{i\,n}^{I}\right)}_{x_{i}}$. For training, we use the classification threshold of 0.6 and add restrictions that the FFN satisfies the marker tendency, which is described in section “Marker Tendency.” The trained FFN is called “*cFFN*_*x_i_*_.” Then, we can use a vector-valued function notation as follows:

$$c\,F\,F\,N=\left(c\,F\,F\,N_{x_{1}},\cdots ,c\,F\,F\,N_{x_{k}}\right),$$

which is called a “cellular state transitional FFN (cFFN).”

### Marker Tendency

We assume that there are marker nodes in the network that can define a state as desired or undesired state. In addition, there is a tendency that the number of desired states in training data decreases from iFFN to tFFN and then to fFFN. However, the number of undesired states in training data increases, which is referred to as marker tendency. We impose restrictions on marker tendency for training iFFN, tFFN, fFFN, and cFFN. We use symbol $\#\,F\,F\,N_{x_{i}}^{\xi }\,\left(s_{i\,n}^{\tau }\right)$ to denote the number of states with active *x*_*i*_ in the output $F\,F\,N_{x_{i}}^{\xi }\,\left(s_{i\,n}^{\tau }\right)$.

#### Toy Network

Node *x*_*6*_ is a unique marker; a state with active or inactive *x*_*6*_ is considered as a desired or undesired state, respectively. Let $\#\,s_{i\,n}^{I}$ and $\#\,s_{t\,a\,r}^{I}$ denote the number of desired states in $s_{i\,n}^{I}$ and $s_{t\,a\,r}^{I}=\left(s_{t\,a\,r,1}^{I},\cdots ,s_{t\,a\,r,I_{k}}^{I}\right)$, respectively. When training $F\,F\,N_{x_{6}}^{1}$, we use a lower bound and an upper bound

$l\,o\,w\,e\,r_{x_{6}}^{I}=\frac{1}{2}\,\min\left\{\#\,s_{i\,n}^{I},\#\,s_{t\,a\,r}^{I}\right\}$ and $u\,p\,p\,e\,r_{x_{6}}^{I}=2\,l\,o\,w\,e\,r_{x_{6}}^{I}$

to add the restriction of marker tendency

$$l\,o\,w\,e\,r_{x_{6}}^{I}\leq \#\,F\,F\,N_{x_{6}}^{1}\,\left(s_{i\,n}^{I}\right)\leq u\,p\,p\,e\,r_{x_{6}}^{I}.$$

Replacing $\left(\#\,s_{i\,n}^{I},\#\,s_{t\,a\,r}^{I}\right)$ with $\left(\#\,s_{i\,n}^{T},\#\,s_{t\,a\,r}^{T}\right)$, we add the restriction of marker tendency when training $F\,F\,N_{x_{6}}^{2}$:

$$l\,o\,w\,e\,r_{x_{6}}^{T}\leq \#\,F\,F\,N_{x_{6}}^{2}\,\left(s_{i\,n}^{T}\right)\leq u\,p\,p\,e\,r_{x_{6}}^{T}.$$

Replacing $\left(\#\,s_{i\,n}^{I},\#\,s_{t\,a\,r}^{I}\right)$ with $\left(\#\,s_{i\,n}^{F},\#\,s_{t\,a\,r}^{F}\right)$ and $\left(\#\,s_{i\,n}^{c\,F\,F\,N},\#\,s_{t\,a\,r}^{c\,F\,F\,N}\right)$, we define

$u\,p\,p\,e\,r_{x_{6}}^{F}=\frac{1}{2}\,\min\left\{\#\,s_{i\,n}^{F},\#\,s_{t\,a\,r}^{F}\right\}$ and $\,u\,p\,p\,e\,r_{x_{6}}^{c\,F\,F\,N}=\frac{1}{2}\,\min\left\{\#\,s_{i\,n}^{c\,F\,F\,N},\#\,s_{t\,a\,r}^{c\,F\,F\,N}\right\}$.

We add the restrictions of marker tendency when training $F\,F\,N_{x_{6}}^{3}$:

$$\#\,F\,F\,N_{x_{6}}^{3}\,\left(s_{i\,n}^{C}\right)\leq u\,p\,p\,e\,r_{x_{6}}^{F},\#\,c\,F\,F\,N_{x_{6}}\,\left(s_{i\,n}^{I}\right)\leq u\,p\,p\,e\,r_{x_{6}}^{c\,F\,F\,N}.$$

#### CACC21

Nodes *x*_*16*_ and *x*_*21*_ are the markers for desired and undesired state, respectively. The state with values (*x*_16_,*x*_21_) = (1,0) or (0,1) is considered to be a desired or undesired state, respectively. Let $\#\,s_{i\,n,x_{16}}^{I}$ and $\#\,s_{t\,a\,r,x_{16}}^{I}$ denote the number of states with active *x*_*16*_ in $s_{i\,n}^{I}$ and $s_{t\,a\,r}^{I}$, respectively. When training $F\,F\,N_{x_{16}}^{1}$, we use a lower bound and an upper bound

$l\,o\,w\,e\,r_{x_{16}}^{I}=\frac{1}{2}\,\min\left\{\#\,s_{i\,n,x_{16}}^{I},\#\,s_{t\,a\,r,x_{16}}^{I}\right\}$ and $u\,p\,p\,e\,r_{x_{16}}^{I}=2\,l\,o\,w\,e\,r_{x_{16}}^{I}$

to add the restriction of marker tendency:

$$l\,o\,w\,e\,r_{x_{16}}^{I}\leq \#\,F\,F\,N_{x_{16}}^{1}\,\left(s_{i\,n}^{I}\right)\leq u\,p\,p\,e\,r_{x_{16}}^{I}.$$

Similarly, we define $\#\,s_{i\,n,x_{21}}^{I}$, $\#\,s_{t\,a\,r,x_{21}}^{I}$, $l\,o\,w\,e\,r_{x_{21}}^{I}=\max\left\{\#\,s_{i\,n,x_{21}}^{I},\#\,s_{t\,a\,r,x_{21}}^{I}\right\}$, and $upper_{x_{21}}^{I}=\frac{3}{2}lower_{x_{21}}{}^{I}$.

We add the restriction of marker tendency when training $F\,F\,N_{x_{21}}^{1}$:

$$l\,o\,w\,e\,r_{x_{21}}^{I}\leq \#\,F\,F\,N_{x_{21}}^{1}\,\left(s_{i\,n}^{I}\right)\leq u\,p\,p\,e\,r_{x_{21}}^{I}.$$

Replacing $\left(\#\,s_{i\,n,x_{16}}^{I},\#\,s_{t\,a\,r,x_{16}}^{I}\right)$ and $\left(\#\,s_{i\,n,x_{21}}^{I},\#\,s_{t\,a\,r,x_{21}}^{I}\right)$ with $\left(\#\,s_{i\,n,x_{16}}^{T},\#\,s_{t\,a\,r,x_{16}}^{T}\right)$ and $\left(\#\,s_{i\,n,x_{21}}^{T},\#\,s_{t\,a\,r,x_{21}}^{T}\right)$, respectively, we add the restrictions of marker tendency when training $F\,F\,N_{x_{16}}^{2}$ and $F\,F\,N_{x_{21}}^{2}$:

$l\,o\,w\,e\,r_{x_{16}}^{T}\leq \#\,F\,F\,N_{x_{16}}^{2}\,\left(s_{i\,n}^{T}\right)\leq u\,p\,p\,e\,r_{x_{16}}^{T}$ and $l\,o\,w\,e\,r_{x_{21}}^{T}\leq \#\,F\,F\,N_{x_{21}}^{2}\,\left(s_{i\,n}^{T}\right)\leq u\,p\,p\,e\,r_{x_{21}}^{T}\,$.

Replacing $\left(\#\,s_{i\,n,x_{16}}^{I},\#\,s_{t\,a\,r,x_{16}}^{I}\right)$ with $\left(\#\,s_{i\,n,x_{16}}^{F},\#\,s_{t\,a\,r,x_{16}}^{F}\right)$ and $\left(\#\,s_{i\,n,x_{16}}^{c\,F\,F\,N},\#\,s_{t\,a\,r,x_{16}}^{c\,F\,F\,N}\right)$, we define

$$\begin{array}{c} l\,o\,w\,e\,r_{x_{16}}^{F}=\frac{1}{2}\,\min\left\{\#\,s_{i\,n,x_{16}}^{F},\#\,s_{t\,a\,r,x_{16}}^{F}\right\},\,u\,p\,p\,e\,r_{x_{21}}^{F}\\ =\max\left\{\#\,s_{i\,n,x_{21}}^{F},\#\,s_{t\,a\,r,x_{21}}^{F}\right\},\\ l\,o\,w\,e\,r_{x_{16}}^{c\,F\,F\,N}=\frac{1}{2}\,\min\left\{\#\,s_{i\,n,x_{16}}^{c\,F\,F\,N},\#\,s_{t\,a\,r,x_{16}}^{c\,F\,F\,N}\right\},\,u\,p\,p\,e\,r_{x_{21}}^{c\,F\,F\,N}\\ =\max\left\{\#\,s_{i\,n,x_{21}}^{c\,F\,F\,N},\#\,s_{t\,a\,r,x_{21}}^{c\,F\,F\,N}\right\} \end{array}$$

and add the restrictions when training $F\,F\,N_{x_{16}}^{3},F\,F\,N_{x_{21}}^{3},c\,F\,F\,N_{x_{16}}$, and *cFFN*_*x*_21__:

$$\begin{array}{c} l\,o\,w\,e\,r_{x_{16}}^{F}\leq \#\,F\,F\,N_{x_{16}}^{3}\,\left(s_{i\,n}^{C}\right),\#\,F\,F\,N_{x_{21}}^{3}\,\left(s_{i\,n}^{C}\right)\leq u\,p\,p\,e\,r_{x_{21}}^{F},\\ l\,o\,w\,e\,r_{x_{16}}^{c\,F\,F\,N}\leq \#\,c\,F\,F\,N_{x_{16}}\,\left(s_{i\,n}^{I}\right),\#\,c\,F\,F\,N_{x_{21}}\,\left(s_{i\,n}^{I}\right)\leq u\,p\,p\,e\,r_{x_{21}}^{c\,F\,F\,N}. \end{array}$$

#### MAPK33

Nodes *x*_*3*_ and *x*_*28*_ are the markers for desired and undesired state, respectively. The state with values (*x*_3_,*x*_28_) = (1,0) or (0,1) is considered to be a desired or undesired state, respectively. Replacing numbers (16, 21) of markers (*x*_16_,*x*_21_) for CACC21 with numbers (3, 28) of markers (*x*_3_,*x*_28_) for MAPK33 provides similar restrictions of marker tendency when training iFFN, tFFN, fFFN, and cFFN.

## Data Availability Statement

The original contributions presented in the study are included in the article/Supplementary Materials, further inquiries can be directed to the corresponding author/s.

## Author Contributions

S-MC and K-HC designed the project, supervised the research, analyzed the results, and co-wrote the manuscript. S-MC and LA performed the modeling and simulation analysis. All authors contributed to the article and approved the submitted version.

## Conflict of Interest

The authors declare that the research was conducted in the absence of any commercial or financial relationships that could be construed as a potential conflict of interest.
