# Supplementary figures and images for: Boolean Feedforward Neural Network Modeling of Molecular Regulatory Networks for Cellular State Conversion
Source: Front Physiol. 2020 Dec 1;11:594151. doi: 10.3389/fphys.2020.594151 (PMC7736109; doi:10.3389/fphys.2020.594151)

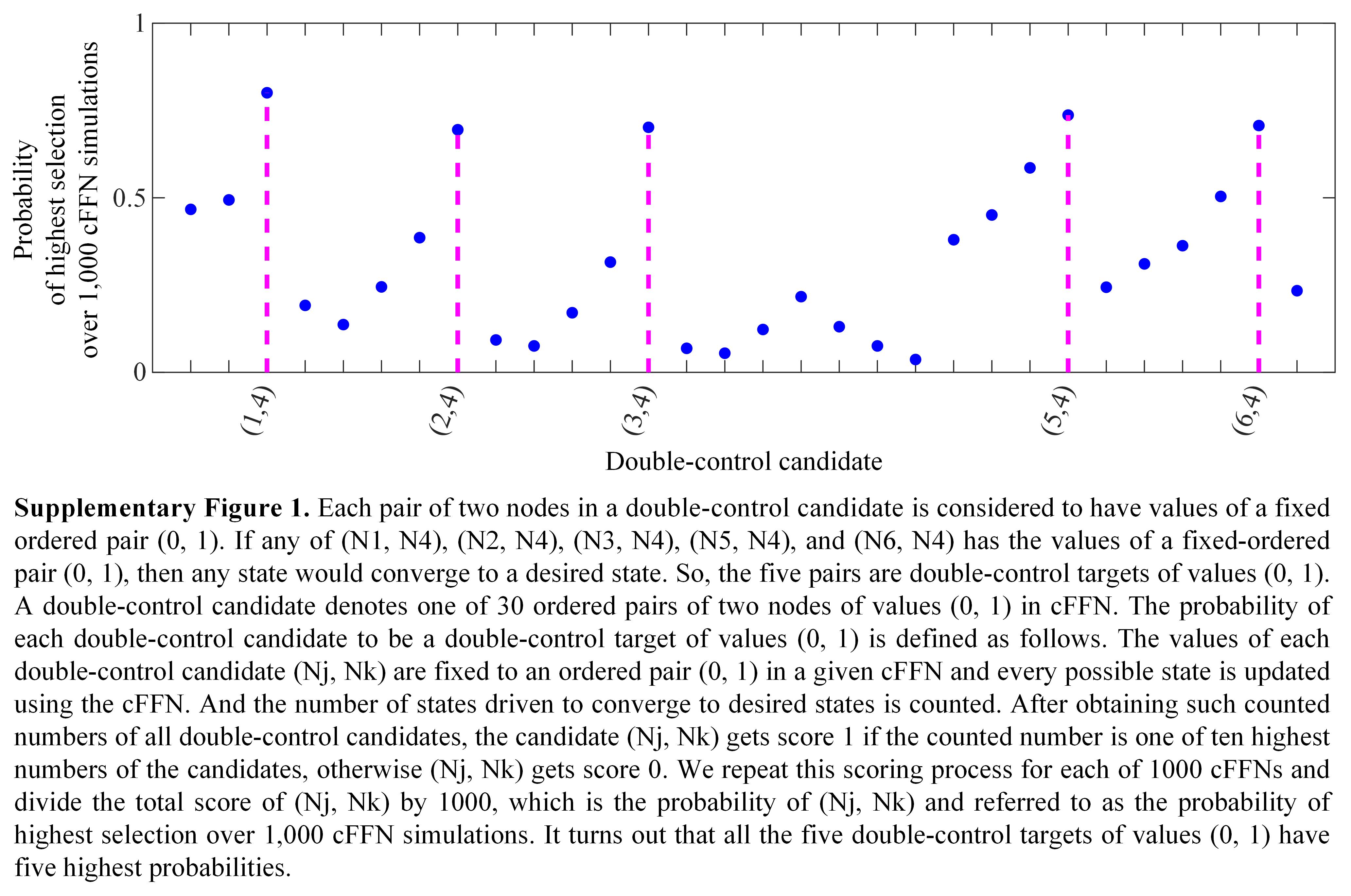

Supplement: Supplementary file 2 [file Image_1.JPEG]

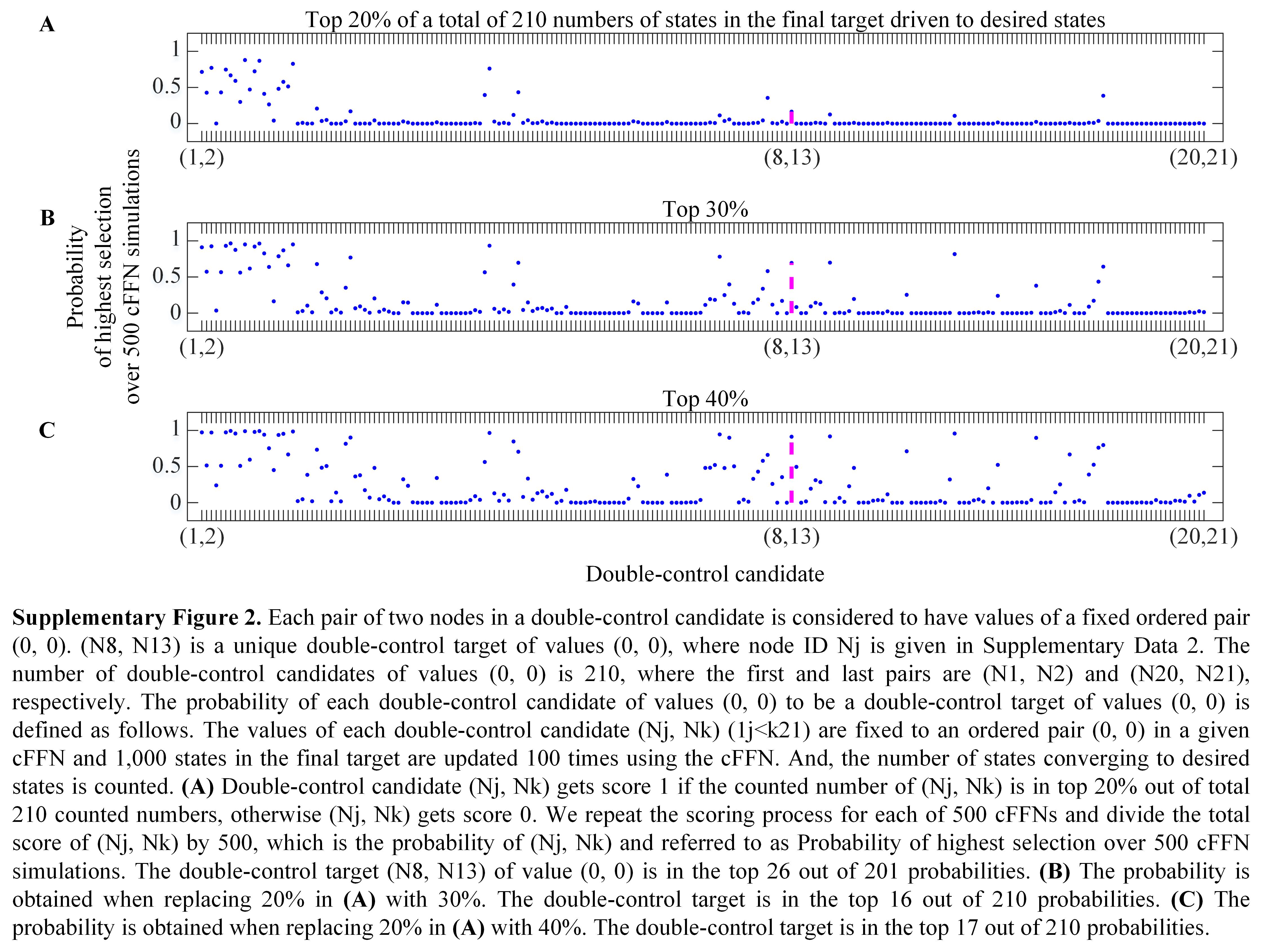

Supplement: Supplementary file 3 [file Image_2.JPEG]

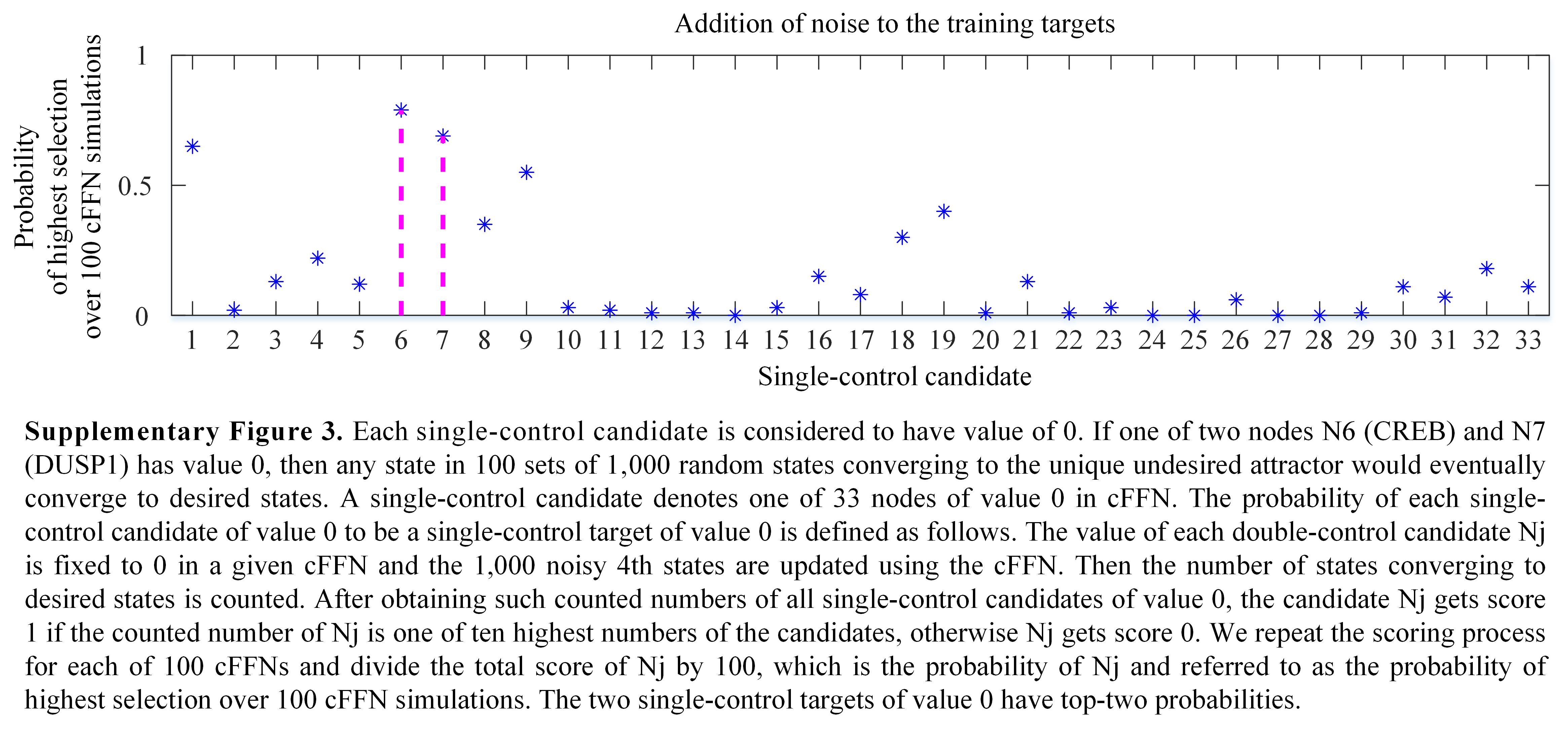

Supplement: Supplementary file 4 [file Image_3.JPEG]

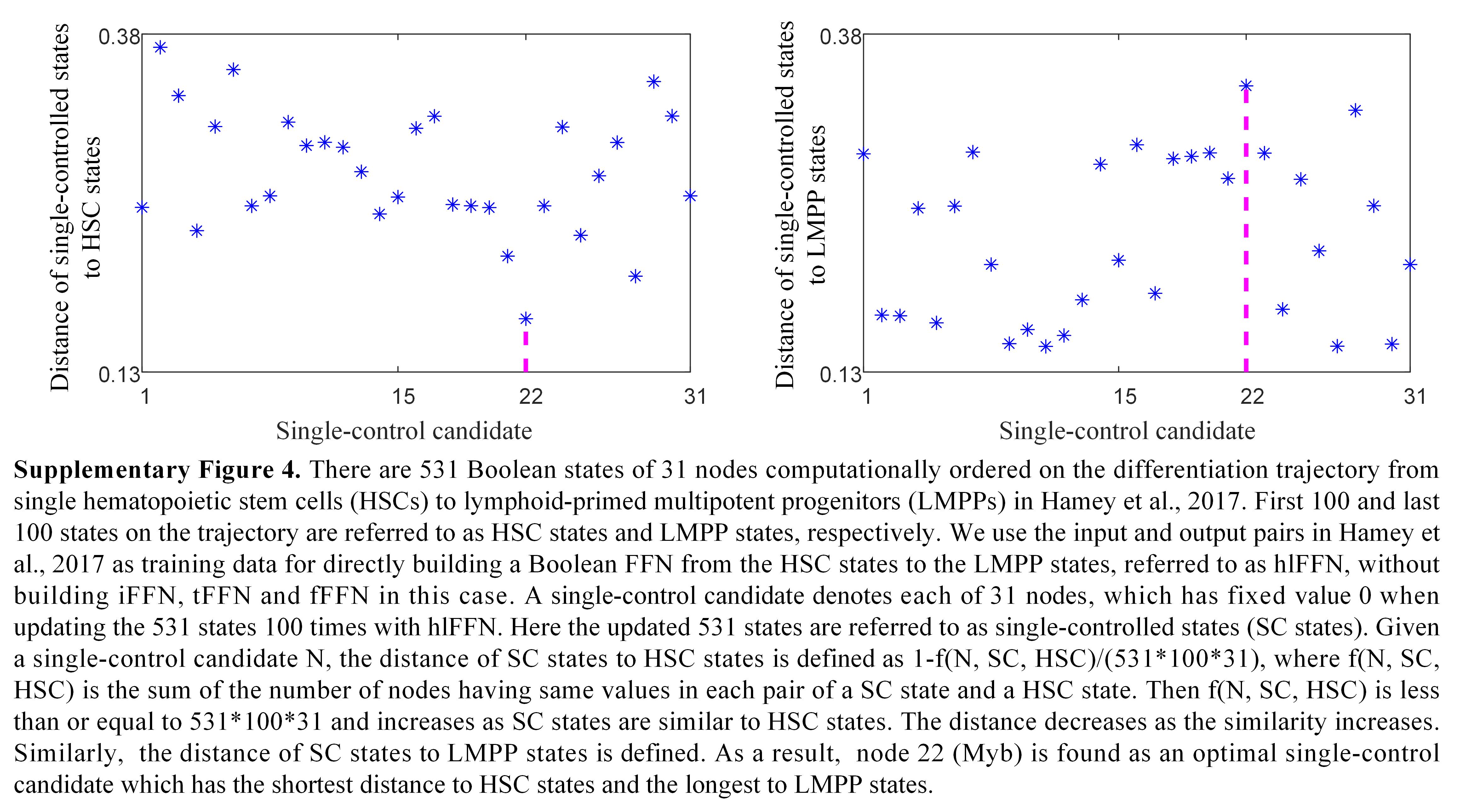

Supplement: Supplementary file 5 [file Image_4.JPEG]
